# Biologically Relevant Dynamical Behaviors Realized in an Ultra-Compact Neuron Model

**DOI:** 10.3389/fnins.2020.00421

**Published:** 2020-05-12

**Authors:** Pablo Stoliar, Olivier Schneegans, Marcelo J. Rozenberg

**Affiliations:** ^1^National Institute of Advanced Industrial Science and Technology (AIST), Tsukuba, Japan; ^2^CentraleSupélec, CNRS, Université Paris-Saclay, Sorbonne Université, Laboratoire de Génie Electrique et Electronique de Paris, Gif-sur-Yvette, France; ^3^Université Paris-Saclay, CNRS, Laboratoire de Physique des Solides, Orsay, France

**Keywords:** spiking neural networks, neuron models, leaky-integrated-and-fire, artificial intelligence, neuromorphic electronic circuits, neuromorphic computers

## Abstract

We demonstrate a variety of biologically relevant dynamical behaviors building on a recently introduced ultra-compact neuron (UCN) model. We provide the detailed circuits which all share a common basic block that realizes the leaky-integrate-and-fire (LIF) spiking behavior. All circuits have a small number of active components and the basic block has only three, two transistors and a *silicon controlled rectifier* (SCR). We also demonstrate that numerical simulations can faithfully represent the variety of spiking behavior and can be used for further exploration of dynamical behaviors. Taking Izhikevich’s set of biologically relevant behaviors as a reference, our work demonstrates that a circuit of a LIF neuron model can be used as a basis to implement a large variety of relevant spiking patterns. These behaviors may be useful to construct neural networks that can capture complex brain dynamics or may also be useful for artificial intelligence applications. Our UCN model can therefore be considered the electronic circuit counterpart of Izhikevich’s (2003) mathematical neuron model, sharing its two seemingly contradicting features, extreme simplicity and rich dynamical behavior.

## Introduction

In his 2003 landmark paper Izhikevich (2003) emphasized that to develop a large-scale model of the brain one faces seemingly mutually exclusive requirements: on one hand the model had to be simple enough to allow for efficient computation and, on the other, it had to be able to produce a rich variety of biologically relevant firing patterns. Interestingly, the same dilemma is encountered for the implementation of neurons in neuromorphic circuits – i.e., circuits that perform computations based on the architecture of the brain. Recently we proposed an ultra-compact neuron (UCN) circuit that realizes the leaky integrate and fire model (Rozenberg et al., 2019). That circuit complies with the first requirement, as it was simply based on only three active devices, two transistors and a thyristor, or SCR, plus a capacitor and a few resistors. Here we shall show that the UCN model also complies with the second requirement. Specifically, we shall demonstrate that the UCN is a circuit block that, with minimal variations, may realize at least 12 out of the 20 biological relevant behaviors highlighted by Izhikevich (2004). To reproduce those behaviors has become a *de facto* standard to demonstrate the relevance of a spiking neuron model implemented on different physical supports. The literature is very diverse and growing fast, so we shall only cite a few examples here and refer the readers to further references in those works and in the review of Indiveri et al. (2011): the digital processor chips TrueNorth developed by IBM (Cassidy et al., 2013; Merolla et al., 2014) and the more recent ODIN by ICTEAM (Frenkel et al., 2019); the compact neuron circuit, with only 14 MOSFET transistors proposed by Wijekoon and Dudek (2008); or the radically different spiking neuron based on vanadium dioxide (Yi et al., 2018), a Mott insulator memristive material (del Valle et al., 2018, del Valle et al., 2019). Other interesting proposals, which aimed at a faithful physical implementation of the Izhikevich mathematical model equations are: a compact circuit of MOS transistors in the subthreshold regime, simulated with MOSIS libraries (Rangan et al., 2010); a CMOS digital neuron for event-driven computation, simulated in Spice (Imam et al., 2010).

### Silicon Neuron (SiN) Circuits

The UCN belongs to the class of SiN circuits (Indiveri et al., 2011), which are electronic hardware implementations of systems that aim to emulate the electric behavior of biological neurons. These SiN blocks may then be integrated to construct larger circuits (Qiao et al., 2015), such as to emulate neural network for artificial intelligence applications, or brain-like systems for basic neuroscience research. The SiN circuits are inspired from a multiplicity of mathematical neuronal models that range from the simplest integrate and fire to the realistic Hodgkin-Huxley (Gerstner et al., 2014). Depending on the desired goal, SiN implementations may favor different features, such as low power dissipation, circuit simplicity, low variability, realistic behavior, tunability, etc. Typically, they are implemented using CMOS technology and VLSI (Indiveri et al., 2011), and they can be broadly classified as sub-threshold or above-threshold depending on the conduction mode of the transistors. The sub-threshold systems follow from the pioneer work of Mead (Mead, 1989, Mead, 1990) and of Mahowald and Douglas (1991) that emphasized the similarity between the exponential behavior of carrier conduction in transistor channels with that of ionic channels in neurons, and coined the concept of “neuromorphic behavior.” The systems in the sub-threshold regime have the additional attractive features of low power dissipation, which follows from the small currents, and time constants that are compatible with the biological ones (Indiveri et al., 2011). However, a main drawback is the so called device mismatch, which is a relatively large variability between cells (Indiveri et al., 2011). As an example of this approach we may mention that of Yu and Cauwenberghs (2010) that implemented a SiN to realize the realistic Hodgkin-Huxley model. The above-thershold implementations avoid mismatch and thus have the precision needed to faithfully recreate the mathematical models that motivate them. These SiN circuits also operate a time-constants that are much faster (10^3^–10^4^) than the biological ones, unless they adopt off-chip larger capacitors. One example of above-threshold systems is the implementation of a tunable Hodgkin-Huxley model by Saighi et al. (2011).

A different approach is to develop SiN circuits that are motivated on generalizations of the simple integrate and fire model (Gerstner et al., 2014). Some examples are the AdEx (Brette and Gerstner, 2005), the Izhikevich (Izhikevich, 2003, 2004) and the Mihalas-Niebur neuron (Mihalas and Niebur, 2009). These models do not necessarily have a biological underpinning as the Hodgkin-Huxley, but nevertheless were shown to capture the relevant spiking patterns observed in biological neurons. Their main attractive is that their relative simplicity allow for more efficient implementations in both software and hardware (Wijekoon and Dudek, 2008; Folowosele et al., 2009; Livi and Indiveri, 2009; Indiveri et al., 2010; Rangan et al., 2010; van Schaik et al., 2010a, b; Qiao et al., 2015).

### The Ultra-Compact Neuron

In this context the UCN that we introduced recently (Rozenberg et al., 2019) opens a different paradigm. Similar to sub-threshold systems and faithful to the concept of neuromorphic engineering, it exploits an intrinsic non-linearity of an electronic device. Namely the threshold switching of the SCR conductance emulates the firing of biological neurons. As we discussed in Rozenberg et al. (2019), this features permits a drastic reduction to the number of components to implement a basic leaky-integrate-and-fire (LIF) SiN. So in this regard it may be considered as belonging to the class of Compact SiN circuits (Indiveri et al., 2011). However an attractive feature of the UCN is that they can be directly interconnected, therefore need not *a priori* require an additional address-event representation off-chip system. Despite the fact that the thyristor was introduced in the very beginnings of semiconductor electronics, it is currently not a conventional CMOS device. Its development in microelectronics is mostly restricted to protection circuits (Ker and Hsu, 2005), which nevertheless demonstrates that there are no *a priori* impediments for its CMOS implementation. The time-constants associated to the switching of a SCR are short, thus in this regard the operation of the UCN follows similar features as the above-threshold SiN as we mentioned above. Therefore, if the goal is to achieve biological time-scales one may need large “membrane” capacitors, hence our UCN should not be considered compact in regard of the wafer real estate.

In the present work we shall describe how the functionality of the basic UCN block can be extended to realize a variety of biologically relevant spiking patterns, without a sacrifice of circuit simplicity. The paper is organized as follows: In section Materials and Methods we shall describe our recently introduced UCN circuit (Rozenberg et al., 2019). We shall demonstrate how the basic behavior of the UCN can be very precisely captured by means of numerical simulations obtained with LTspice (LTspice^®^, 2020 that we validate against actual circuit measured data. Section Results contain the main results of the present work. In the first part, we exploit the simplicity of the simulation package capabilities to explore extensions of the basic UCN circuit block, searching for different types of biologically relevant dynamical behaviors. We shall demonstrate that small variants of a basic circuit allow us to capture at least 12 out of the 20 dynamical behaviors, including some inhibition ones. In the second part of this section, we use the simulation results to achieve the main goal of this work, namely to provide the explicit circuits and measure them to demonstrate that the thyristor-based UCN can realize the complex firing patterns observed in biological systems. Our simulations inform and guide the implementation of the actual electronic circuits that we construct with out-of-the-shelf components. This feature underscores the relative ease for the reproducibility of our work and may prompt other research groups to embark along the present line of work. The circuits details and the list of components are described in the Supplementary Material. In section Discussion we finally discuss some specific technical aspects of our work in regard of different open challenges in the field.

## Materials and Methods

### The Ultra-Compact Neuron Model

In a recent paper (Rozenberg et al., 2019) we introduced the ultra-compact spiking neuron model that only requires two transistors and a thyristor (SCR). We follow the terminology of Indiveri et al. (2011), where a *compact* model refers to a simple electronic circuit with few components. As was argued in Rozenberg et al. (2019), the UCN has a *minimal* number of components, just one capacitor for the *integrate*, one resistor for the *leak*, and one thyristor for the *fire* functionalities. Therefore, one may consider the electronic circuit as an *ultra compact* realization of the LIF neuron. The thyristor is a standard electronic device, often used in high power applications, which consists of a tri-junction *pnpn* device that can be implemented in VLSI (Tong et al., 2012, 2014).

The behavior of a thyristor can be considered as similar to that of a diode where the access to the conduction state is controlled by an adjustable threshold, whose value is set by the voltage at the gate electrode. Once conducting, the SCR remains in a low resistance state till the current becomes smaller than a small holding value I_hold_. Thus, the SCR polarized in direct has *two resistive states*: a high resistance that switches to a low resistance one when the threshold V_th_ is overcome, and the low resistance that switches back to high when the current is below I_hold_. Therefore, one may consider the SCR as a *memristor* since it is a resistive device with a hysteresis or memory effect. In fact, the I-V characteristics of the SCR are qualitatively similar to a type of memristive device with *volatile resistive switching*, which are based on transition metal oxide materials that display Mott insulator-metal transitions (del Valle et al., 2018; Rozenberg et al., 2019). Work along these lines was recently reported in Yi et al. (2018), where a device made of two VO_2_ memristors was conceived to realize a Hodgkin-Huxley type neuron model. Using variants of that basic device, the authors demonstrated a large variety of biologically relevant spiking patterns.

**FIGURE 1:**
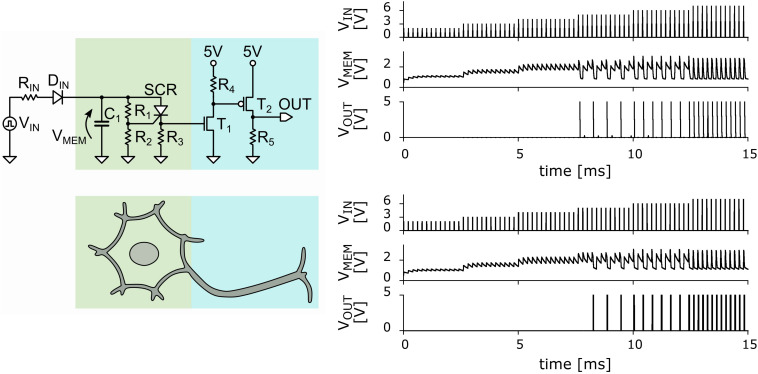
**Left panels:** Basic circuit of the ultra-compact LIF neuron. The portion of the circuit representing the cell body or soma is in green, and that representing the axon is in blue (Rozenberg et al., 2019). **Right panels:** Comparison of the measured (top) and simulated (bottom) basic LIF spiking behavior of the neuron circuit. V_IN_ (measured in volts) denotes the incoming applied voltage pulses from the voltage pulse generator as function of time (in milli-sec). V_MEM_ denotes the voltage at the “membrane” capacitor as function of time. V_OUT_ denotes the output voltage showing a spiking pattern. The existence of a threshold value of V_IN_ for the spiking behavior is clearly observed. It is also observed that as incoming voltage is increased the output spiking rate increases. The agreement between measured data and the simulations is excellent, with the exception of a small transient near the threshold input voltage value.

In Figure 1 we show the schematic circuit of the UCN that can be easily implemented using out-of-the-shelf components. Details of the circuit elements and their values are provided in the Supplementary Material. The memristive effect of the SCR can be straightforwardly exploited in the “soma” block of the UCN circuit (see Figure 1) to achieve the basic LIF behavior. The SCR is initially in a high-resistance state during the *integrate* phase, providing a small *leakage* to the capacitor charge. Then, upon reaching the voltage threshold, the SCR switches to the low-resistance state and *fires* a pulse of current. The current is due to the charge accumulated in the capacitor that rapidly discharges through the SCR. The small holding current ensures the almost full discharge of the capacitor.

The UCN circuit has also a second bock, the “axon” block with two transistors (see Figure 1), which strengthens the current pulse. This feature is key to enable that the output spikes of one (upstream) neuron may excite a second (downstream) neuron. This enables the UCN to be interconnected as modular blocks of a *neural network circuit* as it was shown in a previous publication (Rozenberg et al., 2019).

### Validation of Numerical Simulations of the UCN Circuit

We may exploit the fact that our UCN is simply implemented with standard electronic components to reproduce the spiking behavior using the standard electronic circuit simulation package LTspice, which is freely available (freeware) (LTspice^®^, 2020). In order to represent the actual SCR that we adopted in our circuits, we found convenient to modify the default parameters of the EC103D1 thyristor (SCR) model (LITTELFUSE, 2019). Specifically, we changed the value of the parameter BF from 6.10 to 2.90, and all the other parameters were left unchanged (further details are provided in the Supplementary Material).

The main results of the simulations of the basic UCN block are shown in Figure 1. The validation of the numerical data is done by comparison to the data measured in the actual circuit. As can be observed in the figure, the agreement is excellent. We may note a minor difference in a transient effect when the input reached the threshold voltage to excite spikes. Besides the small difference, the remarkable agreement validates the LTspice package simulations as an efficient method to explore variants of the basic UCN circuit and search for biologically relevant dynamical behaviors. The results of the numerical exploration will then serve us to inform the actual circuit implementations, which is the ultimate goal of our work.

## Results

In this section we present our results. We firstly describe the biologically relevant behaviors obtained through the numerical simulations study of circuits that are variations from the UCN basic block. Then, secondly, we describe the results of the measurements made on actual electronic circuits, whose implementation was informed by the numerical simulations study.

### Numerical Simulation Study

**FIGURE 2:**
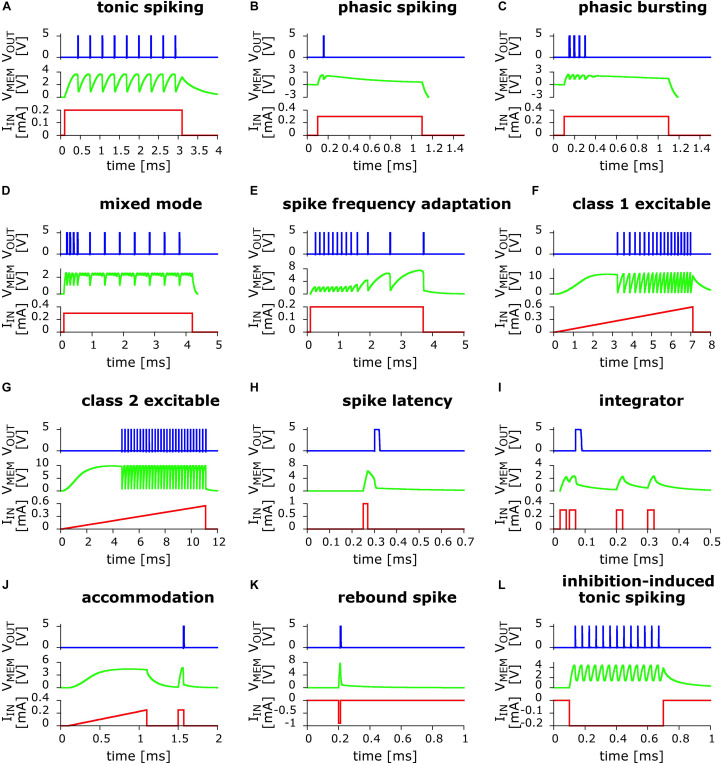
Twelve biologically relevant behaviors obtained from numerical simulations of small variants of the basic UCN LIF circuit, using the LTspice code. In red line is the applied input current excitation I_IN_ as function of time. In green line is the “membrane” voltage V_MEM_ at capacitor C_1_. In blue line is the output voltage V_OUT_ that shows the dynamical spiking behavior. From left to right and top to bottom: **(A)** Tonic spiking; **(B)** phasic spiking; **(C)** phasic bursting; **(D)** mixed (burst then spiking); **(E)** spike frequency adaptation; **(F)** class 1 excitable; **(G)** class 2 excitable; **(H)** spike latency; **(I)** integrator; **(J)** accommodation; **(K)** rebound spike (negative polarity input); and **(L)** inhibition-induced (negative polarity input) tonic spiking.

In Figure 2 we present the results of the exploration of circuits variants. We were able to obtain 12 out of the 20 biologically relevant behaviors captured initially by the Izhikevich neuron model (Izhikevich, 2003, 2004). However, we do not exclude the possibility of capturing the totality of those behaviors, a task that we are leaving for future work.

The behaviors that we obtained include some of the most significant ones. For instance, we find *class 1 and class 2 excitability*, panels (f) and (g), which were originally identified by Hodgkin as the two prototypical ways an individual neuron can start spiking when excited by an external current source (Hodgkin, 1948). In addition, we also obtained the most basic behaviors, such as *phasic spiking and phasic bursting*, Figures 2B,C, which are considered to be associated to a neuron signaling or flagging the beginning of an activity or the presence of a stimulus. Another biologically relevant behavior that we captured is *spike frequency adaptation*, Figure 2E, which is key to habituation. Importantly this behavior is also a key feature of neuronal circuits that can reproduce some basic global brain behaviors, such as asynchronous irregular and regular oscillatory spiking states (Destexhe, 2009; di Volo et al., 2019).

We also obtained *accommodation*, Figure 2J, where the neuron reacts to the rate of change of the input potential. This behavior is associated to a threshold in the time domain, as the neuron gets excited by sudden changes in the environment. It can also be considered as the neuromorphic equivalent of a high-pass filter. We should note, however, that our second input pulse is of the same strength as the first, as small variation with respect to the respective Izhikevich pattern (Izhikevich, 2004). *Delayed phasic spiking* or spike latency was also captured, Figure 2H, which is a behavior that may allow the system to adjust the timing of its reaction to a given input.

The two last behaviors displayed in Figures 2K,L, show that the UCN can also be adapted to accept negative polarity input and produce both, the *rebound spike* and *inhibition-induced tonic spiking*. The negative polarity excitability is associated to inhibition and hyperpolarization of the cell body. For further discussion on the biological relevance of the behaviors we refer the reader to the work of Izhikevich (2004) and the references therein.

All these behaviors were obtained with variants of the basic LIF circuit that involved only one neuron. However, some additional complex behaviors may require the combination of two or more neurons. This interesting possibility lies beyond the scope of the present work.

#### Simulated Circuits

**FIGURE 3:**
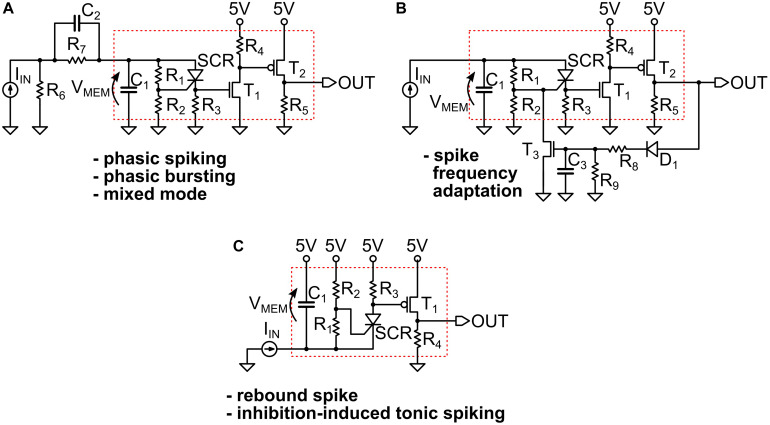
**(A,B)** The circuits that implement the simulated spiking behaviors shown in the Figure 2 of the main text. They all represent small variants of a basic UCN circuit block that is indicated by a red dotted line box. We indicate with an arrow the definition of the “membrane” voltage V_MEM_ on the capacitor C_1_. The circuit adapted for negative input is shown in **(C)**, where we indicate with a red box the basic UCN block that is slightly modified with respect to the original. Note that the rebound spike may also be called inhibition-induced phasic spike. The specific values of the components are provided in Supplementary Material.

In Figure 3 we provide some examples of the simulated circuits. As one can see, they are in fact small variants of the basic LIF model block, which is indicated by a red dotted box in the panels. That portion of the circuit is the UCN block that we introduced and discussed in the previous section and in Figure 1.

Figure 3A, we observe that the small addition made to the basic UCN block is to merely supplement it with an (RC) passive differentiator circuit at the input. This enables to obtain either phasic spiking or phasic bursting behaviors, as the input capacitor gets charged with the first (or few first) incoming pulses and then prevents further DC excitation of the UCN block. On the other hand, in the circuit of Figure 3B we observe that the UCN is supplemented with a feed-back loop. The key feature here is that the feed-back is connected to the gate of the SCR, regulating the effective resistance that grounds the gate. This, in turn, lowers the value of the anode-cathode threshold voltage for the resistive switch of the SCR, and hence one may obtain the spike-frequency adaptation behavior.

As shown in Figure 3C, the implementation of the inhibition induced behaviors can be achieved by simply exchanging the positions of the SCR with R_3_, and of T_1_ with R_4_. In this case, the neuron is excited with negative polarity input pulses. In principle, we no longer need to use the second transistor T_2_ of the axon block, which was only meant to invert the polarity of the outgoing pulse. Therefore, the UCN for inhibition-induced input excitation requires even less components than the original UCN block (red box). However, T_2_ would be required if one desires to generate negative polarity pulses on output.

These simulated circuits will serve as a basis to inform the implementation of the actual electronic circuits that we describe in the next subsection.

### Biologically Relevant Behaviors Realized in Actual UCN Electronic Circuits

**FIGURE 4:**
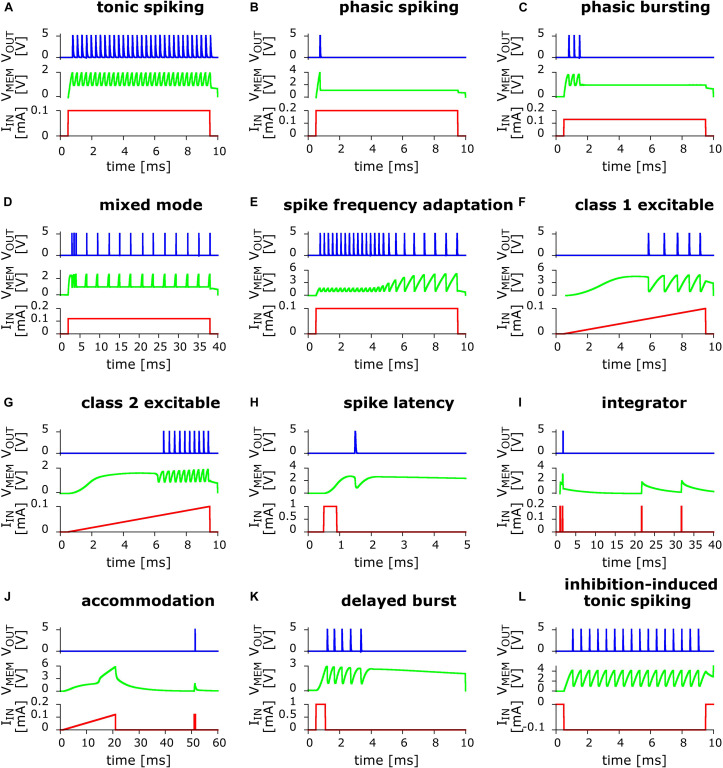
Twelve biologically relevant behaviors measured in small variants of the basic UCN circuit. In red line is the applied input current excitation I_IN_ as function of time. In green line is the “membrane” capacitor voltage V_MEM_. In blue line is the output voltage V_OUT_ that shows the dynamical spiking behavior. From left to right and top to bottom: **(A)** Tonic spiking; **(B)** phasic spiking; **(C)** phasic bursting; **(D)** mixed (burst then spiking); **(E)** spike frequency adaptation; **(F)** class 1 excitable; **(G)** class 2 excitable; **(H)** spike latency; **(I)** integrator; **(J)** accommodation; **(K)** delayed burst (note: this behavior is biologically plausible but not among the Izhikevich set); and **(L)** inhibition-induced (negative polarity input) tonic spiking (note: the “rebound spike” behavior can be trivially obtained from this one via a shorter time input). Further details on the measured circuits along with the values of their components are described in the Supplementary Material.

Our stated strategy was to use simulations to rapidly explore circuit variants, but the ultimate goal is of course to implement the actual electronic circuits. The soundness of this approach is demonstrated by the diversity of the behaviors that we were able to realize, as shown in Figure 4.

Eleven behaviors, shown in Figures 4A–J,L, are close implementations of the previously simulated circuits. They include both, positive and negative input excitation. We note that a twelfth behavior, *rebound spike*, can be trivially obtained from behavior (l) by simply reducing the duration of the input pulse, therefore is not shown. Interestingly, during our experimental circuits study, we also found an additional biologically plausible behavior, shown in Figure 4K that corresponds to *delayed bursting* (Zeldenrust et al., 2018). Other interesting spiking patterns were also observed, including negative input, which we leave to future work.

#### Variations of the UCN Electronic Circuit Block

**FIGURE 5:**
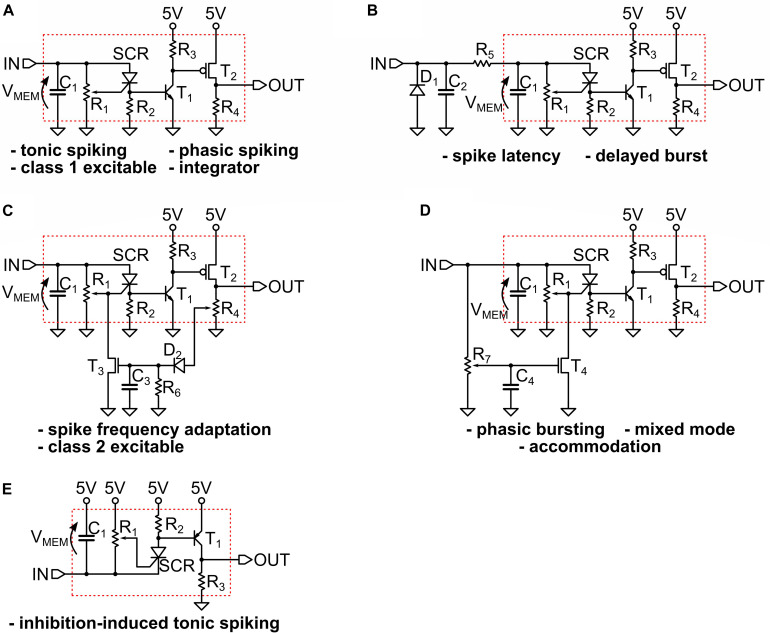
**(A–D)** The circuits that implement the measured spiking behaviors shown in the Figure 3. We indicate with an arrow the definition of the “membrane” voltage V_MEM_ on the capacitor C_1_. The circuit adapted for negative input is shown in **(E)**, where we indicate with a red box the basic UCN block that is slightly modified with respect to the original. The specific values of the components are provided in Supplementary Material.

In Figure 5 we show some significant examples of the actually implemented circuits (the specific values of the components are provided in the Supplementary Material). Similarly, as was the case of numerical simulations, all the circuits are rather small variants of the basic UCN block that we indicate with a red dotted box in the figure.

The excellent agreement between the spiking patterns of the simulations and the actual circuits is quite apparent upon comparison of the many panels of Figures 2, 4. However, we should also mention that while the simulated circuits always provided a good starting point, it was necessary to make further modifications in the actual circuit implementation in order to achieve the desired spiking pattern. One of the most significant differences that illustrates the point is in Figure 5D, which shows the circuit for phasic spiking, mixed mode and accommodation, and can be compared to that of Figure 3A. Actually, both circuits provide valid solutions, however, upon implementation of the simulated 3-a, we found that it imposed too high a demand of current from the input signal generator. Hence, we search for a circuit variant. This was obtained by exploiting the additional freedom of tuning the gate of the SCR. This small example illustrates the benefits and shortcoming of simulations, with respect to the ultimate goal, which is the circuit implementation.

We also note that the implementation of negative input pulses (inhibition), shown in Figure 5E, also required some small modifications. The rest of the circuits are almost identical to the simulated ones. Besides the small circuit changes, it was also sometimes necessary to adapt the input strengths, such as comparing (Figure 2F) and (Figure 4F); or different spiking frequency were obtained, such as in Figures 2G, 4G. In any case, we want to emphasize that exact agreement was not the goal, except in the initial validation of the numerical model described in Section Materials and Methods. For the whole variety of obtained behaviors the differences remained merely quantitative, and within an order of magnitude, and the qualitative agreement was always satisfactory.

Some additional details on the choice of electronic components and the implementation of the measurement system are given in the Supplementary Material.

## Discussion

In this work we have illustrated the versatility of the UCN circuit to capture a significant number of biologically relevant neuronal behaviors. We have not attempted to demonstrate the totality of the 20 behaviors identified by Izhikevich (2004). Rather, our goal was to demonstrate that the minimal UCN circuit is a sound basis to implement a new type of spiking neuron model of remarkable simplicity. Another important result of our study was the successful implementation of the numerical simulation package to efficiently search for the complex spiking patterns. This required the implementation of the SCR model. More generally, reliable numerical simulations methods become an essential tool to implement small neural sub-circuits counting tens or hundreds of spiking neurons.

The key feature that enables this circuit simplicity is the memristive behavior of the SCR, which is a conventional electronic component that may be implemented in CMOS technology (Ker and Hsu, 2005; Tong et al., 2012). However, we should also note that although the UCN model and the extensions proposed in the present work only require a reduced number of electronic components, they are of different types, which may pose a challenge for the integration into a single technology. Nevertheless, this may be achieved by Bi-CMOS (Alvarez, 1990), or the more recent BCD8sP technology (Roggero et al., 2013). Simulation of our UCN circuits to implement actual chips is an exciting prospect that is beyond the scope of the present work.

Also in regard of the prospects for microelectronic implementation, one should be aware that, similarly to all compact neuron model circuits based on standard electronics, the UCN also requires a “membrane” capacitor to integrate charge. This feature remains a significant problem for miniaturization as the capacitors still require a relatively large physical space in VLSI.

The ultimate solution for a low-power and low-footprint spiking neuron device may therefore require memristors based on Mott materials (del Valle et al., 2018). However, achieving a reliable control and a theoretical understanding of the metal-insulator transitions in those compounds still represent a significant challenge (Yi et al., 2018; del Valle et al., 2019).

To conclude, the UCN model is a simple modular block that can be used to implement spiking neuron circuits. The present work demonstrates that its simplicity does not prevent the realization of complex spiking patterns, beyond the integrate and fire paradigm.

Our work opens a new way for the implementation of large neuronal networks with biological plausibility and of unprecedented simplicity.

## Data Availability StatemeNt

All the information required to obtain the datasets generated for this study is included in the article/Supplementary Material.

## AuThor Contributions

All authors listed have made a substantial, direct and intellectual contribution to the work, and approved it for publication.

## Conflict of Interest

The authors declare that the research was conducted in the absence of any commercial or financial relationships that could be construed as a potential conflict of interest.
