# Supplementary material for: Biologically Relevant Dynamical Behaviors Realized in an Ultra-Compact Neuron Model
Source: Front Neurosci. 2020 May 12;14:421. doi: 10.3389/fnins.2020.00421 (PMC7247826; doi:10.3389/fnins.2020.00421)
Supplement: Supplementary file 1 [file Data_Sheet_1.PDF]

frontiers

Supplementary Material

1. Components

1.1 Components for the basic UCN block

In the table below, we provide the values of the components used for the basic UCN circuit that is shown in Fig. 1.

Component	Value (simulated circuit)	Value (implemented circuit)
SCR	custom model (see methods)	CR02A
T1	IRLML6246	IRLML6246
T2	BSS84	ZVP3310A
D_{IN}	1N4148	1N4148
C1	15nF	15nF
R1	68kΩ	68kΩ
R2	390kΩ	390kΩ
R3	1.5kΩ	1.5kΩ
R4	10kΩ	10kΩ
R5	4.7kΩ	4.7kΩ
R_{IN}	1.5kΩ	1.5kΩ

Supplementary Table 1. List of components for circuits simulated with the LTspice and implemented, corresponding to the data of Fig.1 of Main Text.

1.2 Components for the numerically simulated circuits

In the table below, we provide the values of the components used for each one of the circuit variants that are shown in Fig.3 of Main Text.

Component	Panel of Fig.3 (main text)	Value
SCR	a, b, c	Custom model (see Methods)
T1	a, b	IRLML6246
T1	c	standard model (BSS84)
T2	a, b	standard model (BSS84)
T3	b	IRLML6246
D1	b	standard model (1N4148)
C1	a (phasic spiking)	3nF
C1	a (phasic bursting)	3nF
C1	a (mixed mode)	5nF
C1	b	10nF
C1	c	1nF
C2	a (phasic spiking)	5nF
C2	a (phasic bursting)	15nF

C2	a (mixed mode)	17nF
C3	b	20nF
R1	a (phasic spiking)	50kΩ
R1	a (phasic bursting)	50kΩ
R1	a (mixed mode)	40kΩ
R1	b	40kΩ
R1	c	100kΩ
R2	a (phasic spiking)	300kΩ
R2	a (phasic bursting)	300kΩ
R2	a (mixed mode)	30kΩ
R2	b	40kΩ
R2	c	100kΩ
R3	a (phasic spiking)	1.5kΩ
R3	a (phasic bursting)	1.5kΩ
R3	a (mixed mode)	1kΩ
R3	b	0.5kΩ
R3	c	3kΩ
R4	a (phasic spiking)	10kΩ
R4	a (phasic bursting)	10kΩ
R4	a (mixed mode)	50kΩ
R4	b	10kΩ
R4	c	20kΩ
R5	a	10kΩ
R5	b	5kΩ
R6	a	30kΩ
R7	a (phasic spiking)	none
R7	a (phasic bursting)	none
R7	a (mixed mode)	11kΩ
R8	b	20kΩ
R9	b	300kΩ

Supplementary Table 2. List of components for circuits simulated with the LTspice code that realize the spiking behaviors shown in Fig.2 of the main text (circuits are in Fig.3).

<u>1.3 Components for the actually implemented circuits</u>

In the table below, we provide the values of the components used for each one of the circuit variants that are shown in Sup. Fig. 2.

**Component**	**Panel of Fig.5 (main text)**	**Value**
SCR	a, b, c, d, e	P0118MA
T1	a, b, c, d	2N3904
T1	e	2N3906

T2	a, b, c, d, e	ZVP3310A
T3	c	ZVN0124A
T4	d	ZVN0124A
D1	b	1N4148
D2	c	1N4148
C1	a, b, c, d, e	10nF
C2	b	47nF
C3	c	100nF
C4	d	100nF
R1	a, b, c, d, e	500kΩ (25-turn trimmer)
R2	a, b, c, d, e	2kΩ
R3	a, b, c, d, e	100kΩ
R4	a, b, d, e	100kΩ
R4	c	100kΩ (25-turn trimmer)
R5	b	100kΩ
R6	c	100kΩ
R7	d	100kΩ (25-turn trimmer)

Supplementary Table 3. List of components for the actual circuits that were implemented and measured to obtain the spiking behaviors shown in Fig.4 of the Main Text (circuits are in Fig.5)

2. Details on the actual circuit implementation and measurements

One main difference between the simulated and the implemented circuits is the transistor T_1 (Fig.5 in the Main Text) sensing the voltage at the SCR cathode. We envisage the use of a low-threshold NMOS transistor, as done in the simulations. However, we were not able to get it commercially in a no-SMD package. Therefore, in the actual circuit implementation we adopted for T_1 a standard NPN transistor.

In some circuits we replaced the simulated resistor by a trimmer resistor. This was done solely for practical convenience.

There are also differences in the implementations of the actual circuit and the simulation one in the case of mixed mode behavior [see in Main Text panel (b) of Fig.3 and panel (d) of Fig.5]. Both circuits are actually valid solutions. We nevertheless favored the chosen circuit implementation as we find it less demanding to the input signal generator.

The measurements were done using a National Instrument PCIe-6343 Multifunction I/O Device (ADQ board with 32 AI (16-Bit, 500 kS/s), 4 AO (900 kS/s), and 48 DIO Multifunction I/O). We also developed an ad-hoc ADQ software based on National Instrument LabVIEW. Since the output of the PCIe-6343 are voltage outputs, we implemented a V-to-I converter based on an Analog Devices LT1168 Instrumentation Amplifier. We also buffered the analog inputs using Texas Instruments TL084 Operational Amplifiers.

3. LTSpice model for the SCR

```
.SUBCKT EC103D1     1       2       3
*           TERMINALS:      A       G       K
Qpnp        6       4       1       Pfor    OFF
Qnpn        4       6       5       Nfor            OFF
Rfor        6       4       5G
Rrev        1       4       5G
Rshort      6       5       1MEG
Rlat        2       6       9.09
Ron         3       5       513.4m
Dfor        6       4       Zbrk
Drev        1       4       Zbrk
Dgate       6       5       Zgate
.MODEL      Zbrk    D       (IS=3.2E-16   IBV=100U  BV=400)
.MODEL      Zgate   D       (IS=1E-16    IBV=100U  BV=10     VJ=0.3)
.MODEL      Pfor    PNP     (IS=5E-15    BF=2.90  CJE=5p    CJC=2p    TF=0.3U)
.MODEL      Nfor    NPN     (IS=1E-12    ISE=1E-9  BF=10.0    RC=0.45    CJE=30p    CJC=2p    TF=0.3U)
.ENDS
```
